# Circulating Lipopolysaccharides and Impaired Antioxidant Status in Patients With Atrial Fibrillation. Data From the ATHERO-AF Study

**DOI:** 10.3389/fcvm.2021.779503

**Published:** 2021-11-17

**Authors:** Danilo Menichelli, Roberto Carnevale, Cristina Nocella, Vittoria Cammisotto, Valentina Castellani, Simona Bartimoccia, Giacomo Frati, Pasquale Pignatelli, Daniele Pastori

**Affiliations:** ^1^Department of Clinical, Internal, Anesthesiological and Cardiovascular Sciences, Sapienza University of Rome, Rome, Italy; ^2^Department of Medical-Surgical Sciences and Biotechnologies, Sapienza University of Rome, Latina, Italy; ^3^Department of Angio-Cardio-Neurology, Istituto di Ricovero e Cura a Carattere Scientifico (IRCCS) Neuromed, Località Camerelle, Pozzilli, Italy; ^4^Mediterranea Cardiocentro, Naples, Italy

**Keywords:** atrial fibrillation, lipopolysaccharides, glutathione peroxidase, superoxide dismutase, antioxidant, gut, cardiovascular events

## Abstract

**Objectives:** Atrial fibrillation (AF) is characterized by an oxidative imbalance, which is associated with an increased risk of cardiovascular events (CVEs). It is unclear whether low grade endotoxemia may contribute to the impaired antioxidant status in AF patients. We investigated the relationship between circulating lipopolysaccharides (LPS) and antioxidant status in AF patients.

**Patients and Methods:** *Post-hoc* analysis from the ongoing prospective observational cohort ATHERO-AF study including 907 patients. Antioxidant status was evaluated by the activity of glutathione peroxidase 3 (GPx3) and superoxide dismutase (SOD). Patients were divided into two groups to evaluate the risk of CVEs: (1) LPS below median and GPx3 above median (*n* = 254); (2) LPS above median and GPx3 below median (*n* = 263).

**Results:** The mean age was 73.5 ± 8.3 years, and 43.1% were women. Median LPS and GPx3 were 50.0 pg/ml [interquartile range (IQR) 15–108] and 20.0 U/ml (IQR 10.0–34.0), respectively. Patients of Groups 2 were older, with a higher prevalence of heart failure. LPS above the median was associated with reduced GPx3 [Odds Ratio for LPS 1.752, 95% Confidence Interval (CI) 1.344–2.285, *p* < 0.001] and SOD (OR 0.525, 95%CI 0.403–0.683) activity after adjustment for CHA_2_DS_2_VASc score. In a mean follow-up of 54.0 ± 36.8 months, 118 CVEs occurred, 42 in Group 1 and 76 in Group 2 (Log-Rank test *p* = 0.001). At multivariable Cox regression analysis, Group 2 was associated with a higher risk of CVEs [Hazard Ratio (HR) 1.644, 95%CI 1.117–2,421, *p* = 0.012], along with age ≥ 75 years (HR 2.035, 95%CI 1.394–2.972, *p* < 0.001), diabetes (HR 1.927, 95%CI 1.280–2.900, *p* = 0.002), and previous cerebrovascular disease (HR 1.895, 95%CI 1.251–2.870, *p* = 0.003) and previous cardiovascular disease (HR 1.708, 95%CI 1.149–2.538, *p* = 0.008).

**Conclusions:** Our study indicates that circulating LPS may contribute to impaired antioxidant status in patients with AF. Patients with coincidentally high LPS and reduced GPx3 activity showed the highest risk of CVEs.

## Introduction

A clinically relevant incidence of cerebrovascular and cardiovascular complications is still detectable in patients with atrial fibrillation (AF) despite optimal anticoagulant treatment (1–3). This may be due to several reasons including both a suboptimal integrated prevention strategy for the management of comorbidities (4), as shown by data indicating that only a minority of patients with AF are currently managed according to the guidelines-recommended “Atrial fibrillation Better Care” (ABC) pathway (5). In addition, the presence of non-traditional risk factors which may contribute to this residual cardiovascular risk has been demonstrated, for which specific prevention strategies are not yet established. In this context, oxidative stress, endotoxemia and low-grade inflammation have been the most widely investigated in patients with cardiovascular disease and in the AF population (6–8). Oxidative imbalance seems to be particularly important for the onset and maintenance of AF (9, 10) as also shown by interventional trials showing a lower incidence of new-onset AF in patients supplemented with antioxidants (11, 12). Increased oxidative stress in AF may be the result of the up-regulation of pro-oxidant enzymes, such as NADPH oxidase, or the down-regulation of circulating antioxidant systems such as glutathione peroxidase 3 (GPx3) and superoxide dismutase (SOD), leading to an increased concentration of reactive oxidant species. Markers of oxidative stress seem to have a prognostic role for cardiovascular events (CVEs) in AF (13).

Regarding endotoxemia, gut microbiota and its derivatives, such as lipopolysaccharides (LPS) and trimethylamine N-oxide, are increasingly recognized as contributing factors for systemic atherosclerosis and its complications through several pathogenetic mechanisms (14, 15). For instance, LPS may have a pro-thrombotic effect by activating the Toll-like receptor 4 (TLR4) pathway (16). The TLR4 is the receptor for Gram-negative bacterial endotoxin, localized in several cells, including macrophages (17), platelets and myocytes (18). A recent experimental study showed that LPS may have pro-arrhythmic effects. Indeed, LPS-treated pericardium from rabbits had higher expression of pro-inflammatory cytokines, and LPS favored atrial arrhythmogenesis through the interaction of TLR4, as shown by treatment with a TLR4 inhibitor (19).

In addition, LPS may contribute to systemic inflammation also in other cardiovascular diseases such as heart failure (HF). Thus, HF is associated with gut barrier dysfunction resulting in an increased LPS translocation into systemic circulation (20). Also in this case, TLR4 knockout mice had lower inflammation, confirming the role of LPS-TLR4 interaction as an important mechanism contributing to systemic inflammation (21).

In addition to gut barrier dysfunction, gut microbiome alterations have been recognized as important pathogenetic factors in the onset and progression of several cardiovascular diseases (22).

Previous experimental evidence showed that LPS injection increases the short-term biosynthesis of selenium dependent GPx as a mechanism of defense against the LPS-induced oxidative stress in rat hepatic endothelial and Kupffer cells (23). However, it is unclear whether LPS may modulate the activity of antioxidant enzymes also in humans and what is the effect of long-term exposure to low-grade endotoxemia. We hypothesized that chronic low-grade LPS may contribute to the impairment of antioxidant status in patients with AF. At this aim, we investigated the association between circulating gut-derived LPS and the activity of the GPx3, the blood isoform of GPx involved in the detoxification of superoxide anion, in patients with AF enrolled in the ATHERO-AF study cohort.

## Methods

### Study Cohort

We performed a prospective, observational cohort study, which included patients from the ongoing ATHERO-AF cohort at “Sapienza” University of Rome. All patients were treated with oral anticoagulants, either vitamin K antagonists (VKAs) or direct oral anticoagulants (DOACs), the latter since their introduction into the market in Italy in 2013. During the first clinical examination a complete personal medical history was collected, including drug therapy and comorbidities. Definitions of cardiovascular risk factors have been previously reported (24).

Patients were not included if they had prosthetic heart valves, or the presence of any severe valvulopathies, severe cognitive impairment, chronic infectious (HIV, HCV, HBV) or autoimmune systemic disease. Furthermore, subjects were excluded from the study if they had active neoplastic diseases or liver insufficiency (e.g., cirrhosis). Patients were also asked to collect a blood and urine sample. This was not mandatory for all patients.

### Measurement of GPx3 Activity

GPx3 activity was measured in serum by Assay Kit (Abcam). In this assay, GPx reduces the probe Cumene Hydroperoxide while converting reduced glutathione to its oxidized form, glutathione disulfide. The generated oxidized glutathione is converted into reduced glutathione by glutathione reductase, using nicotinamide adenine dinucleotide phosphate as a reducing agent. GPx3 activity is proportional to the decrease in the absorbance of nicotinamide adenine dinucleotide phosphate at 340 nm. One unit of GPx is defined as the amount of enzyme that will cause the oxidation of 1 nmole of nicotinamide adenine dinucleotide phosphate (from NADPH to NADP+) per minute at 25C. GPx activity was expressed as U/ml. Intra- and inter-assays were 4.0 and 6.0% (25).

### Measurement of SOD Activity

Extracellular SOD (SOD3) activity was assessed by a colorimetric activity kit (Arbor Assay) in serum samples. Samples were incubated with the substrate followed by xanthine oxidase reagent. The xanthine oxidase generates superoxide in the presence of oxygen, which converts a colorless substrate into a colored product. The colored product was read at 450 nm. SOD activity was expressed as U/ml. Intra- and inter-assays were 4.6 and 6.1%, respectively.

### Circulating Lipopolysaccharides

Lipopolysaccharides serum levels were measured using a commercial ELISA kit (Cusabio, Wuhan, China). Standards of LPS, purified from Escherichia coli, and blood samples were plated for 2 h at room temperature onto a microplate precoated with the antibody specific for LPS. After incubation, samples were read at 450 nm. Values were expressed as picograms per milliliter; intra-assay and interassay coefficients of variation were 8 and 10%, respectively. The coefficient of variation was <10% (26).

### Follow-Up and Cardiovascular Events

Follow-up was performed by periodic clinical evaluations in the outpatient clinic for INR check in patients on VKAs and for DOAC monitoring, or by telephone (during the pandemic period). The primary outcome of the study consists in the evaluation of CVEs. CVEs was defined as fatal/non-fatal ischemic stroke (IS), fatal/non-fatal myocardial infarction (MI), cardiac revascularization/coronary bypass surgery, and cardiovascular death, as previously described (24).

The study was approved by the local ethical board of Sapienza and conducted according to Principles embodied in the Declaration of Helsinki.

### Statistical Analysis

Categorical variables were reported as number and percentages which were compared by the Pearson chi-squared test. Mean and standard deviation or median and interquartile range were used for continuous variables, which were compared by Student T or Mann-Whitney *U*-test, respectively. Logistic regression analysis was used to investigate the association between LPS and reduced GPx3 activity (dependent variable, below median). We divided the cohort into two groups according to the median value of LPS and GPx3 to compare baseline clinical characteristics: Group 1 low LPS (below median), high GPx3 (above median); Group 2: high LPS (above median), low GPx3 (below median). We estimated the cumulative risk for CVEs of each group using a Kaplan-Meier method. The survival curves were then formally compared using the log-rank test. Cox proportional hazards analysis was used to calculate the adjusted relative hazard ratios [HR] and 95% Confidence Intervals [CI] of CVEs by each clinical variable.

All tests were two-tailed and only *p*-values < 0.05 were considered as statistically significant. The analyses were performed using SPSS-25.0 software (IBM, Armonk, NY).

## Results

The mean age of the whole cohort (*n* = 907 patients) was 73.5 ± 8.3 years, and 43.1% were women. The mean CHA_2_DS_2_-VASc was 3.6 ± 1.5 and median LPS, GPx3 and SOD were 50.0 pg/ml [interquartile range (IQR) 15–108], 20.0 U/ml (IQR 10.0–34.0) and 2.2 U/ml (IQR 1.5–3.2), respectively (Table 1). In the whole cohort, LPS above the median was associated with reduced GPx3 activity at univariable logistic regression analysis [Odds Ratio (OR) 1.758, 95%CI 1.351–2.287, *p* < 0.001] and after adjustment for CHA_2_DS_2_VASc score (OR for LPS 1.752, 95%CI 1.344–2.285, *p* < 0.001).

**Table 1:** Clinical characteristics of the ATHERO-AF cohort.

	**Whole cohort (*n* = 907)**
Age (years)	73.5 ± 8.3
Age ≥ 75 years (%)	45.3
Female sex (%)	43.1
Arterial hypertension (%)	89.7
Diabetes (%)	20.0
Heart Failure (%)	17.2
Previous cerebrovascular disease (%)	14.6
Previous cardiovascular disease (%)	24.8
CHA_2_DS_2_-VASc (mean)	3.6 ± 1.5
Statins (%)	41.8
Proton pump inhibitors (%)	46.6
Antiplatelets (%)	19.5
Angiotensin converting enzyme inhibitors/angiotensin receptor blocker (%)	69.9
Beta blockers (%)	40.5
Median lipopolysaccharides (pg/ml) (25th-75th percentile)	50.0 (15–108)
Median glutathione peroxidase 3 (U/ml) (25th-75th percentile)	20.0 (10.0–34.0)
Median superoxide dismutase (U/ml) (25th-75th percentile)	2.2 (1.5–3.2)

Similarly, high LPS was associated with reduced SOD activity (multivariable OR 0.525, 95%CI 0.403–0.683).

### Groups of LPS and GPx3

We investigated the effect of concomitant presence of low LPS and High GPx3 on CVEs; **for this reason**, we divided the cohort into two groups according to the median value of LPS and GPx3.

Overall, 254 patients had serum LPS levels below median and serum GPx3 levels above median (Group 1) and 263 patients had serum LPS levels above median and serum GPx3 levels below median (Group 2). Clinical characteristics of the two groups are reported in Table 2.

**Table 2:** Clinical characteristics of patients according to LPS and GPx3 serum levels.

	**Group 1: Low LPS, high GPx3 (*n* = 254)**	**Group 2: High LPS, low GPx3 (*n* = 263)**	***P*-value**
Age (years)	72.5 ± 8.2	74.8 ± 8.1	0.001
Age ≥ 75 years (%)	40.9	51.3	0.022
Female sex (%)	42.1	44.9	0.530
Arterial hypertension (%)	87.8	91.6	0.150
Diabetes (%)	19.3	19.4	0.977
Heart Failure (%)	14.6	23.3	0.012
Previous cerebrovascular disease (%)	14.2	17.5	0.302
Previous cardiovascular disease (%)	24.8	28.9	0.294
CHA_2_DS_2_-VASc (mean)	3.5 ± 1.5	3.9 ± 1.4	0.001
Statins (%)	41.5	41.8	0.952
Proton pump inhibitors (%)	47.4	48.6	0.781
Antiplatelets (%)	21.3	20.2	0.756
ACE-I/ARB	68.1	72.2	0.304
Beta blockers (%)	38.6	41,8	0.452

Patients of Groups 2 were older, had a higher CHA_2_DS_2_ VASc score and had a higher prevalence of heart failure, but no difference was found regarding the prevalence of hypertension, diabetes, previous cardiovascular and cerebrovascular diseases (Table 2).

### CVEs

**Figure 1:**
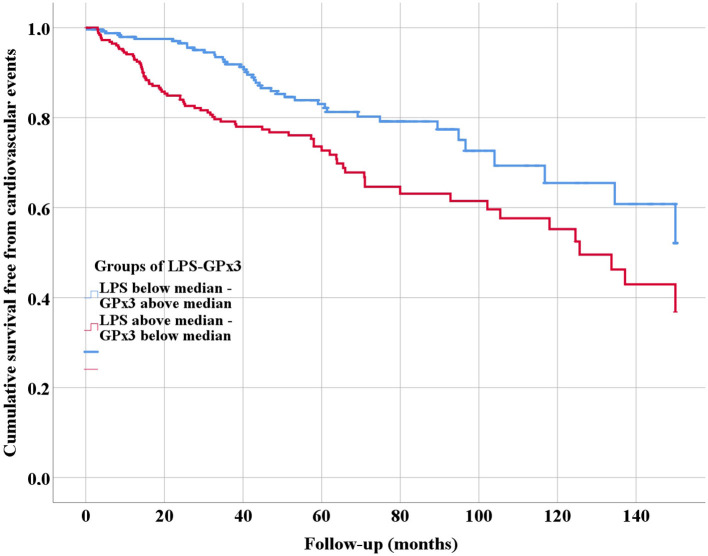
Kaplan-Meier survival curves of cardiovascular events, according to serum levels of lipopolysaccharides (LPS) and glutathione peroxidase 3 (GPx3).

Patients were followed for a mean of 54.0 ± 36.8 months. During follow-up, 118 CVEs occurred. Of these, 42 occurred in Group 1 and 76 in Group 2. At univariable analysis, Group 1 was associated with a lower risk of CVEs compared to Group 2 (Log-Rank test *p* = 0.001) as showed in Figure 1.

At multivariable Cox regression analysis (Table 3), Group 2 was independently associated with a higher risk of CVEs (HR 1.644, 95%CI 1.117–2,421, *p* = 0.012). Other factors associated with higher risk of CVEs were: age ≥ 75 years (HR 2.035, 95%CI 1.394–2.972, *p* < 0.001), diabetes (HR 1.927, 95%CI 1.280–2.900, *p* = 0.002), previous cerebrovascular disease (HR 1.895, 95%CI 1.251–2.870, *p* = 0.003) and previous cardiovascular disease (HR 1.708, 95%CI 1.149–2.538, *p* = 0.008).

**Table 3:** Multivariable Cox regression analysis of risk factors for cardiovascular events.

	**Hazard ratio**	**95% confidence intervalLow High**	***P*-value**
Low GPx3 and High LPS (vs. high GPx3 and low LPS)	1.644	1.117–2.421	0.012
Female sex	0.990	0.675–1.451	0.959
Age ≥ 75 years	2.035	1.394–2.972	<0.001
Arterial hypertension	1.117	0.512–2.441	0.781
Diabetes	1.927	1.280–2.900	0.002
Heart failure	1.345	0.888–2.036	0.162
Previous cerebrovascular disease	1.895	1.251–2.870	0.003
Previous cardiovascular disease	1.708	1.149–2.538	0.008

*Gpx3, glutathione peroxidase 3; LPS, lipopolysaccharides*.

## Discussion

In this cohort study, we found a significant association between low-grade endotoxemia and impaired antioxidant activity in patients with AF. Patients with concomitant low activity of GPx3 and high circulating levels of LPS showed the highest risk for CVEs.

Our study adds to previous studies suggesting a role for LPS as a pro-oxidant and pro-atherogenic factor contributing to cardiovascular disease. Indeed, LPS has been shown to be involved into multiple pathways leading to an increased production of reactive species of oxygen (ROS) in different cardio-metabolic diseases (27). A previous study that analyzed the composition of carotid atherosclerotic plaque showed that the positivity for LPS and Toll-like receptor 4 (TLR4) was higher coincidentally with activated macrophages (28). Furthermore, LPS induced a dose-dependent TLR4-mediated Nox2 up-regulation by human monocytes, an increased oxidized LDL (ox-LDL) cholesterol and hydrogen peroxide production (28).

Our finding that LPS inversely correlated with GPx3 and SOD activity, which are among the most important circulating antioxidant enzymes, suggests that LPS may not only increase the activity of pro-oxidant enzymes but it may also potentially exert a negative regulation of the antioxidant ones. However, we do not know if this may be related to a direct effect of LPS on the antioxidant enzymes, or if their down-regulation may be mediated by oxidative compounds. At this regard, experimental studies showed that bovine epithelial cells treated with scalar doses of LPS showed a significant reduction of antioxidant enzymes activity, such as GPx3, SOD and catalase (29). Similarly, a study investigating intestinal oxidative stress in piglets challenged with LPS, showed a decrease of SOD, catalase and GPx activity, which was blunted by probiotic supplementation (30). Furthermore, considering that an aging-related decline in the activity of GPx enzymes has been previously reported (31, 32), it is also conceivable that the oxidative damage related to LPS may be even more evident in elderly patients with a high atherothrombotic burden (33).

Of note is that LPS and GPx3 were associated with an increased risk of CVEs, independently from age and traditional cardiovascular risk factors. This lead to hypothesize that biomarkers of oxidative stress may be useful to identify patients with a residual cardiovascular risk (34). However, the possibility of measuring the activity of these enzymes is not widely available in clinical practice.

Clinical therapeutic implications of our study rely on the possibility of reducing circulating levels of LPS or in preventing its related oxidative damage. On one hand, it would be interesting to test whether the modulation of gut microbiota composition by nutritional intervention or non-absorbable antibiotic administration, aimed at reducing LPS translocation into the systemic circulation would result in a lower systemic oxidative stress and improved antioxidant status.

In a previous study, we showed an inverse association between the adherence to Mediterranean diet and circulating LPS in AF patients, with legumes and fruits consumption showing the strongest inverse association (26). This association has also been reported in other cardio-metabolic diseases, such as in patients with diabetes (35). However, interventional trials to confirm this finding in patients with AF are needed.

Among pharmacological interventions, a previous metanalysis including patients with liver cirrhosis showed that rifaximin or norfloxacin treatment reduced serum LPS-binding protein (36, 37). In addition, as LPS may also induce mitochondrial dysfunction resulting in an increased ROS production; indeed, mitochondria targeted strategies have been proposed (38).

Statins may represent another therapeutic option as they may reduce pro-oxidant products such as ox-LDL and reduce the activity of oxidant enzymes such as Nox2 (39). Previous evidence also showed that statins may improve some blood antioxidant compounds such as vitamin E (40) and up-regulate GPx activity in a cancer model (41).

In addition to LPS circulating levels, alterations in gut microbiota composition in patients with AF ma provide useful information, and gut modulation may be an interesting complementary therapeutic approach to explore.

As the measurement of GPx3 and SOD is not wide available, and both these enzymes are involved in the detoxification of hydrogen peroxide, we developed a simple method to assess antioxidant ability of plasma to scavenge this compound. The antioxidant status evaluated through this method was also associated with cardiovascular events in the same cohort of AF patients (42).

Our study has some limitations. First, the observational design could not detect a cause-effect relationship but only an association between increased LPS, reduced GPx3 activity and CVEs. Then, we included only Caucasian AF patients, and we could not extend our findings to other populations.

In conclusion, our study adds another piece to the complex scenario on the role of low-grade endotoxemia in favoring a pro-thrombotic phenotype in AF patients. Strategies aimed at reducing LPS may improve antioxidant status and reduce cardiovascular risk in these patients.

## Data Availability Statement

The datasets presented in this article are not readily available because the raw data supporting the conclusions of this article will be made available by the authors upon reasonable request. Requests to access the datasets should be directed to daniele.pastori@uniroma1.it.

## Ethics Statement

The studies involving human participants were reviewed and approved by Ethical Board of Sapienza University of Rome. The patients/participants provided their written informed consent to participate in this study.

## Author Contributions

DM: conception or design of the work, analysis of data, and draft of the manuscript. RC: conception or design of the work, acquisition, interpretation of data for the work, and draft of the manuscript. CN: conception or design of the work, acquisition, interpretation of data for the work, and draft of the manuscript. VCam, VCas, and SB: acquisition, interpretation of data for the work, and draft of the manuscript. GF and PP: interpretation of data for the work, draft of the manuscript, and critically revised the manuscript. DP: analysis of data, critically revised the manuscript, and guarantor of the paper. All authors contributed to the article and approved the submitted version.

## Conflict of Interest

The authors declare that the research was conducted in the absence of any commercial or financial relationships that could be construed as a potential conflict of interest.

## Publisher's Note

All claims expressed in this article are solely those of the authors and do not necessarily represent those of their affiliated organizations, or those of the publisher, the editors and the reviewers. Any product that may be evaluated in this article, or claim that may be made by its manufacturer, is not guaranteed or endorsed by the publisher.
